# Semiparametric modelling of diabetic retinopathy among people with type II diabetes mellitus

**DOI:** 10.1186/s12874-022-01794-4

**Published:** 2023-01-09

**Authors:** Bezalem Eshetu Yirdaw, Legesse Kassa Debusho

**Affiliations:** 1 Department of Statistics, University of South Africa, c/o Christiaan de Wet Road & Pioneer Avenue, Florida 1709 Johannesburg, South Africa; 2 Department of Statistics, University of South Africa, c/o Christiaan de Wet Road & Pioneer Avenue, Private Bag X6, Florida 1710 Johannesburg, South Africa

**Keywords:** Covariate by factor interaction, Diabetes mellitus, Diabetic retinopathy, Semiparametric model, Tensor product interaction

## Abstract

**Background:**

The proportion of patients with diabetic retinopathy (DR) has grown with increasing number of diabetes mellitus patients in the world. It is among the major causes of blindness worldwide. The main objective of this study was to identify contributing risk factors of DR among people with type II diabetes mellitus.

**Method:**

A sample of 191 people with type II diabetes mellitus was selected from the Black Lion Specialized Hospital diabetic unit from 1 March 2018 to 1 April 2018. A multivariate stochastic regression imputation technique was applied to impute the missing values. The response variable, DR is a categorical variable with two outcomes. Based on the relationship derived from the exploratory analysis, the odds of having DR were not necessarily linearly related to the continuous predictors for this sample of patients. Therefore, a semiparametric model was proposed to identify the risk factors of DR.

**Result:**

From the sample of 191 people with type II diabetes mellitus, 98 (51.3%) of them had DR. The results of semiparametric regression model revealed that being male, hypertension, insulin treatment, and frequency of clinical visits had a significant linear relationships with the odds of having DR. In addition, the log- odds of having DR has a significant nonlinear relation with the interaction of age by gender (for female patients), duration of diabetes, interaction of cholesterol level by gender (for female patients), haemoglobin A1c, and interaction of haemoglobin A1c by fasting blood glucose with degrees of freedom $3.2, 2.7, 3.6, 2.3 \, \text{ and }\, 3.7$, respectively. The interaction of age by gender and cholesterol level by gender appear non significant for male patients. The result from the interaction of haemoglobin A1c (HbA1c) by fasting blood glucose (FBG) showed that the risk of DR is high when the level of HbA1c and FBG were simultaneously high.

**Conclusion:**

Clinical variables related to people with type II diabetes mellitus were strong predictive factors of DR. Hence, health professionals should be cautious about the possible nonlinear effects of clinical variables, interaction of clinical variables, and interaction of clinical variables with sociodemographic variables on the log odds of having DR. Furthermore, to improve intervention strategies similar studies should be conducted across the country.

**Supplementary Information:**

The online version contains supplementary material available at 10.1186/s12874-022-01794-4.

## Introduction

Diabetic retinopathy (DR) is one of the microvascular complications of diabetes mellitus that occurs as a result of long-term accumulated damage to the small blood vessels in the retina [1–4]. It has been one of the major cause of blindness in the world [1, 5, 6]. In 2018, around 1 million people were blind due to DR [1]. The estimated annual incidence and progression of diabetic-related eye disease ranged from 2.2% to 12.7% and 3.4% to 12.3%, respectively [4, 7]. There was a noticeable reduction in the number of blindness and vision loss in the world. However, the percentage of blindness and moderate to severe vision impairment due to DR increased by approximately 50% and 53%, respectively [8]. Further, the proportion of DR in Africa ranges from 7% to 62.4%, of which severe DR was observed in 15% of the patients. Ethiopia is one of the first four countries with a high percentage (3.8%) of adult diabetic community in sub-Saharan Africa [9, 10]. A study reveal that the prevalence of retinopathy among people with type II diabetes mellitus and in a group without diabetes was 34.6% and 8.8%, respectively [11].

Studies across the world have shown that, the most predictive factors of diabetic retinopathy are socio-demographic and clinical variables such as age, diabetic duration, lipid profiles, hyperglycaemia and microalbuminuria of a patient [4, 12–19]. Hussain et al. [15] reported that, gender and clinical variables have significant relationships with DR. Furthermore, glycaemic control and body-mass index have significant associations with DR [3, 4]. The landmark studies show that high blood pressure and hyperglycaemia are associated with development and progression of DR [18, 19]. A study from Ethiopia reported that, gender, haemoglobin a1c (HbA1c) and hypertension are predictive risk factors of DR [17]. Some studies have also revealed that the odds of having DR is higher for a patient with higher HbA1c [3, 12, 15, 20], longer duration of diabetes [4, 12] and hypertensive patients [3, 4, 17]. A study based on data from a meta-analysis of seven cohort studies reported that insulin treatment has significant association with DR in patients with type II diabetes mellitus [13]. Another study based on 5.2 years follow up data indicates that variability of fasting plasma glucose (FPG) is a significant predictor of DR [21]. Ten years follow up study also showed that, as compared to patients without DR, patients with DR had a higher level of FPG and HbA1c [22]. A study shows that there is a strong connection between HbA1c and FBG in a diabetic subject [23]

It was illustrated that exploratory data analysis is the initial step that must be done before undertaking any complex statistical procedure [24, 25]. Further, it is the act of looking into the data that helps to understand the variables in the data and the relationship between them. It also helps to determine if the statistical model that is going to be considered for data analysis is appropriate [26, 27]. However, most studies on DR [3, 12, 13, 15, 20–22] skip this essential step of data analysis. Moreover, studies in Ethiopia also used a parametric model, e.g., generalized linear model (GLM) which only identify the linear relationship between the link function and covariates to determine predictive factors of DR without exploring the data [4, 14, 17]. However, because of the incorrect functional form of the model, some high risk covariates may be interpreted as having no relationship with DR.

There are few studies that were conducted on DR at Black Lion Hospital (BLH) [17, 28–30]. These studies identified some socio demographic and clinical variables as predictors of DR. However, in these studies, almost all continuous predictors were categorized and considered as factors, and linear association between the response and predictors was considered via the logit link. However, categorization of continuous variables leads to loss of information and reduces the statistical power to detect the relationship between predictor and response [31]. Therefore, the main aim of this research was to identify the contributing risk factors of DR among people with type II diabetes mellitus at BLH and to estimate the data driven relationship between clinical variables, specifically continuous predictors and DR using semiparametric models. As there is no reported result, at least in Ethiopian situation, on the nonlinear interaction effects of clinical variables and gender on DR, this study was also motivated to assess the nonlinear interaction effects of clinical variables with gender on the log odds of having DR.

## Methodology

### Study area and data

For the current study, we used the same data from Shibru, Aga and Boka [17]. The data is a secondary data that was obtained from Black Lion Hospital (BLH). The hospital is located in Addis Ababa, Ethiopia and it is the largest teaching and referral hospital in Ethiopia. The diabetic unit at BLH gives a service provision for more than 200 individuals per week. For this study, a cross-sectional study design was used. The data was collected from March to April 2018 and all people with type II diabetes mellitus who had a follow up at BLH diabetic unit within the study period were eligible for this study. The study excludes critically ill patients who were very weak to give informed consent to participate in the study.

The sample size was determined using a simple random sampling formula [32] based on a 5% level of significance, 13% prevalence of DR which was obtained from previous study done in the country [33] and 0.05 degree of precision. Further, a 10% non-response rate was considered to get a final sample size of 191 patients for the study. The response variable, DR is a categorical variable with two outcomes (patient with DR and patient without DR) which is measured via direct retinal photographs with Topcon camera [34]. The retinal photographs with a Topcon camera were done by the nurses who had training in DR screening. In the current study, patients with mild non-proliferative DR (NPDR) with occasional haemorrhages; moderate NPDR with moderate intraretinal haemorrhages, soft exudates, and occasional intraretinal microvascular anomalies; severe NPDR with numerous peripheral retinal haemorrhages and/or moderate intraretinal microvascular anomalies and/or definite venous bleedings; proliferative DR (PDR) with new vessels on the disc or elsewhere on the retinal; and macular oedema diagnosed from the presence of hard exudates within one disc diameter of the foveola were considered as DR. Therefore, a patient with any type of DR or having one of these characteristics in one of the two eyes or both eyes was considered as DR. Socio-demographic and treatment related variables were collected via face-to-face individual interview, and clinical variables were extracted from patient’s records. To sum up, this study includes categorical and continuous variables as predictors of DR, where gender, hypertension, insulin treatment, and frequency of clinical visits were considered as factors, and age, duration of diabetes, total cholesterol level, HbA1c and FBG were considered as covariates. In this study, a patient is considered as hypertensive if two different days measurements of systolic and/or diastolic blood pressure are $\ge 140 \,mmHg$ and $\ge 90\, mmHg$, respectively [35].

#### Semiparametric model for binary response

**Fig. 1:**
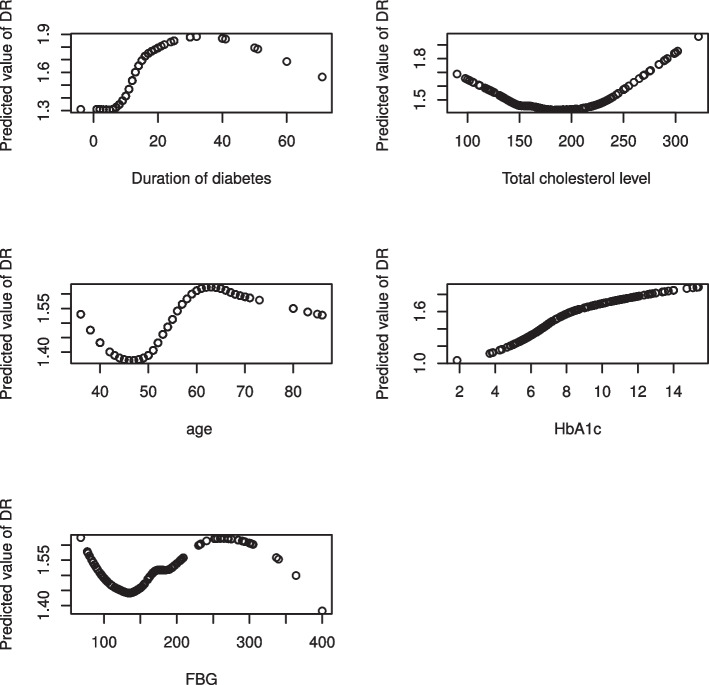
A loess fit to the log odds of diabetic retinopathy and continuous predictors separately

Given the exploratory plots in Fig. 1, a semiparametric model is more reasonable for this data rather than assumptions based restrictive parametric models. Let a binary outcome variable $y_{i}$ denotes the DR status of the $i^{th}$ patient, where $y_{i}=1$ represents patient with DR and $y_{i}=0$ represents patient without DR, let $z_m$ denotes the $m^{th}$ categorical variable, $m=1,\cdots ,M$ and let $x_j$ denotes $j^{th}$ continuous variable, $j=1,\cdots ,J$ then a semiparametric model for the outcome $y_{i}$ is given by:

1 $$\begin{aligned} \begin{array}{lll} g(\mu _i)= \alpha _0+\sum\nolimits_{m=1}^{M} \sum\nolimits_{l=1}^{L_m}\alpha _{ml}z_{iml} +h_{j}(x_{ij})+f_{z_{i}}(x_{ij})+f_{ab}(x_a,x_b), \end{array} \end{aligned}$$

where $\mu _{i}= E(y_i)$, $\alpha _0$ is the model constant, $\sum _{m=1}^M \alpha _{ml}z_{iml}$ is the parametric term of the model for the categorical variables (gender, hypertension, insulin treatment and frequency of clinical visit), $z_{iml}$ is the $l^{th}$ level of $m^{th}$ categorical variable measured on the $i^{th}$ patient and $\alpha _{ml}$ is the corresponding parameter, *M* is total number of categorical variables, $L_{m}$ is number of categories/level of the $m^{th}$ categorical variable, $l=1,\cdots ,L_m$, e.g., when the $m^{th}$ categorical variable has two levels, we have one $\alpha _{ml}$, i.e. $L_{m}=1$ because the first category is treated as a reference category. For example, in this study frequency of clinical visit has three categories (every 1 month, every 3 month and every 6 month), where, every 1 month was treated as a reference category. Additionally, $h_{j}(x_{ij})$ is a smoothing function for the continuous clinical predictors, $f_{z_{i}}(x_{ij})$ is a smoothing function for the covariate by factor level interaction, $x_{ij}$ is the $j^{th}$ continious predictor measured on the $i^{th}$ patient and $f_{ab}(x_a,x_b)$ is a smoothing function for the tensor product interaction of two continuous clinical variables $x_a$ and $x_b$. In a semiparametric model, for each level of a factor we have one curve representing a covariate by factor interaction. For example, in the current study we have age by gender interaction which have two separate curves for male and female. To do this define:

$$z_i \in \{1, \cdots , L_{m}\}$$ and

$$\begin{aligned} \begin{array}{ll} z_{il} = \left\{ \begin{array}{lc} 1,~~~~ \text {if}&{} z_{i}=l \\ 0, &{}else . \end{array}\right. \end{array} \end{aligned}$$

Thus, the model in Expression (1) can be written as:

2 $$\begin{aligned} \begin{array}{llll} g(\mu _i)&{}=&{} \alpha _0+\sum\nolimits_{m=1}^M \sum\nolimits_{l=1}^{L_m}\alpha _{ml}z_{iml} + \beta _{1j}x_{ij}+\beta _{2j}x_{ij}^2 + \cdots +\beta _{pj}x_{ij}^p \\ &{}+&{}\sum\nolimits_{k=1}^{K}b_{kj}(x_{ij}-\kappa _{kj})_+^p \\ &{}+&{} \sum\nolimits_{l=2}^{L_m}z_{il}(\gamma _{0l}+\gamma _{1lj}x_{ij} + \gamma _{2lj}x_{ij}^2 + \cdots +\gamma _{plj}x_{ij}^p) \\ &{}+&{} \sum\nolimits_{l=1}^{L_m}z_{il}\{\sum\nolimits_{k=1}^{K}c_{kj}^{l}(x_{ij}-\kappa _{kj})_+ + f_{ab}(x_a,x_b,) \end{array} \end{aligned}$$

$$\begin{aligned} \begin{array}{l} f_{ab}(x_a,x_b)=\sum\nolimits_{s_1=0}^{p}\sum\nolimits_{s_2=0}^{p} \delta _{s_{1}s_{2}}x_{ia}^{s_1} x_{ib}^{s_2} +\sum\nolimits_{k_{1}=1}^{K_1}\sum\nolimits_{k_{2}=1}^{K_2} b_{k_{1}k_{2}}(x_{ia}x_{ib}-\kappa _{k_{1}k_{2}})^p_+, \end{array} \end{aligned}$$

$w_+=max\{0,w\}$, $\beta _{1j}, \beta _{2j}, \cdots , \beta _{pj}$ are fixed effect parameters for the main effect smoothing functions, $(\gamma _{0l}, \gamma _{1lj},\cdots ,\gamma _{plj})$ are fixed effect parameters for the smoothing function of an interaction of $x_j$ by $L_m$ levels of a factor $z_i$, $x_{ia}$ and $x_{ib}$ are two continuous predictors measured on the $i^{th}$ patient which are considered to have a tensor product interaction effect on the response, $\sum _{s_1=0}^p\sum _{s_2=0}^p \delta _{s_1s_2}$ are fixed effect parameters for the tensor product smoothing interaction $x_a \odot x_b$. Finally, $\kappa _{kj}$ are knots where the $p^{th}$ degree spline evaluated at a covariate $x_{j}$ and covariate by factor interaction of the smoothing term, and $\kappa _{k_{1}k_{2}}$ are knots where the $p^{th}$ degree spline evaluated at the tensor product $x_a \odot x_b$ for the tensor product interaction of the smoothing term, $\sum _{k=1}^{K}b_{kj}(x_{ij}-\kappa _{kj})_+^p$ is the over all smooth term for the main effect, $\sum _{l=1}^{L_m}z_{il}\{\sum _{k=1}^{k}c_{kj}^{l}(x_{ij}-\kappa _{kj})_+\}$ is the deviation from the over all smooth term of the covariate by factor interaction and $\sum _{k_{1}=1}^{K_1}\sum _{k_{2}=1}^{K_2} b_{k_{1}k_{2}}(x_{ia}x_{ib}-\kappa _{k_{1}k_{2}})^p_+$ is the overall smooth term for the tensor product smoothing function. According to [36], a penalized cubic regression spline allows to retain the good properties of splines and has good computational efficiency. Therefore, we have considered a penalized cubic regression spline $(p=3)$ to model nonlinearity of the covariates. The respective random effect coefficients $b_{kj}$, $c_{kj}^l$ and $b_{k_{1}k_{2}}$ were assumed to follow a gaussian distribution, i.e. $b_{kj} \sim N(0,\sigma _{bj}^2)$, $c_k^l \sim N(0, \sigma _{cl}^2)$ and $b_{k_{1}k_{2}}\sim N(0,\sigma ^{2}_{b_{ab}})$, respectively.

#### Proposed semiparametric models

**Fig. 2:**
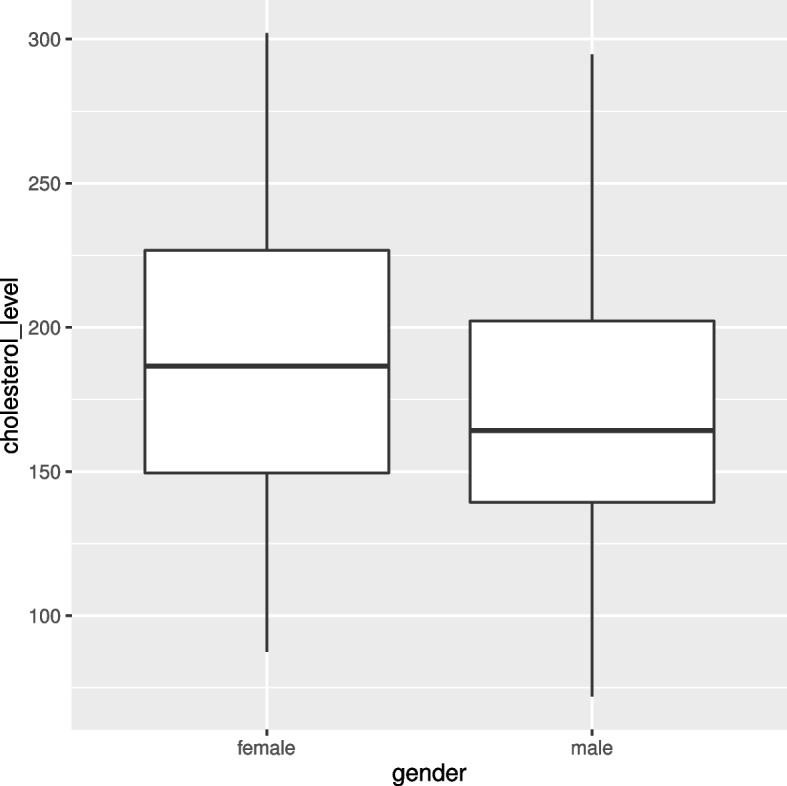
A Boxplot for cholesterol level by gender

In this study, we used exploratory data analysis, such as loess plot and box plot to understand the characteristics of variables and explore the relationship between variables in the data. The locally estimated scatter-plots smoothing presented in Fig. 1 suggest that the relationship between the log odds of having DR and each of the continuous clinical variables is nonlinear. Hence, logistic regression model may be too restrictive to analyse this data. Therefore, semiparametric model is a reasonable choice for this sets of data. Moreover, according to [37], the functional form of a covariate in additive model varies across groups defined by levels of categorical variables. Further, the interaction between age and gender of a diabetic patient is epidemiologically plausible for consideration [38]. Therefore, this study consider the nonlinear interaction of age by gender. A study using a logit link reported that the interaction between mean HbA1c and FBG variability has no significant association with the odds of having DR [21]. However, since both HbA1c and FBG has a nonlinear relationship with the log odds of DR, the interaction of HbA1c and FBG may have a significant nonlinear effect on the log odds of having DR. Moreover, Fig. 2 revealed that there is a variation between the total cholesterol levels of male and female. Therefore, it is worthy to investigate the interactions of age by gender, cholesterol level by gender and HbA1c by FBG ($HbA1c \times FBG$). Thus, we proposed five different semiparametric models. We start with a more general model $(M_1)$ which includes gender, hypertension, frequency of clinical visit (FCV) and insulin treatment (IT) as a linear term and interactions of age by gender, $HbA1c \times FBG$, total cholesterol level (CL) by gender, and duration of diabetes (DD) as nonlinear terms and $M_1$ therefore defined as:

3 $$\begin{aligned} \begin{array}{llll} g(\mu _i) &{}=&{} \beta _0 + \beta _1 \, {gender} +\beta _2 \, {hypertension}+\beta _3 \, {IT}\\ &{}+&{} \beta _4\,FCV + f_{gender}({age})+ f({DD})+f(HbA1c) \\ &{}+&{} f(FBG) + f({HbA1c},{FBG}) +f_{{gender}}({CL}) \end{array} \end{aligned}$$

where, using Expression (2) presentation, for example

$$\begin{aligned} f(DD_{i})&=\beta_{0}+\beta_{1}(DD_{i})+\beta_{2}(DD_{i})^{2} + \ldots +\beta_{p}(DD_{i})^{p}\\ &\quad+\sum\nolimits_{k=1}^{K}b_{k}((DD_{i})-\kappa_{k})_{+}^{p}\end{aligned}$$

and

$$\begin{aligned} \begin{array}{lll} f(HbA1c_i, FBG_i) &{}=&{} \sum\nolimits_{s_1=0}^{p}\sum\nolimits_{s_2=0}^{p} \delta _{s_{1}s_{2}}(HbA1c_i)^{s_1} (FBG_i)^{s_2} \\ &{}+&{} \sum\nolimits_{k_{1}=1}^{K_1}\sum\nolimits_{k_{2}=1}^{K_2} b_{k_{1}k_{2}}((HbA1c_i)(FBG_i)-\kappa _{k_{1}k_{2}})^p_+. \end{array} \end{aligned}$$

The second model $M_2$ was proposed to test the nonlinearity of $HbA1c \times FBG$ and it is given by

4 $$\begin{aligned} \begin{array}{llll} g(\mu _i) &{}=&{} \beta _0 + \beta _1 \, {gender} +\beta _2 \, {hypertension} + \beta _3 \,{IT} \\ &{} +&{} \beta _4\,FCV + \beta _5 \,{HbA1c} + \beta _6\,{FBG} +\beta _7\,{HbA1c}\times {FBG} \\ &{}+&{} f_{gender}(age)+ f({DD}) + f_{{gender}}({CL}) \end{array} \end{aligned}$$

The third model $M_3$ was proposed to test the nonlinearity of age by gender interaction and it is given by

5 $$\begin{aligned} \begin{array}{llll} g(\mu _i) &{}=&{} \beta _0 + \beta _1 \, {gender} + \beta _2 \,{age} + \beta _3\,{age}\times {gender} \\ &{}+&{} \beta _4 \,{hypertension}+ \beta _5 \, {IT} +\beta _6\,FCV \\ &{}+&{} f({DD}) + f({HbA1c},{FBG}) +f_{{gender}}({CL}) \end{array} \end{aligned}$$

The fourth model $M_4$ was proposed to test the nonlinearity of total cholesterol level by gender interaction and it is given by

6 $$\begin{aligned} \begin{array}{llll} g(\mu _i) &{}=&{} \beta _0 + \beta _1 \, {gender} + \beta _2 \,{CL}+ \beta _3\,{CL}\times {gender} \\ &{}+&{} \beta _4 \, {hypertension} + \beta _5 \, {IT}+ \beta _6\,FCV \\ &{}+&{} f_{gender}(age) + f({DD}) + f({HbA1c},{FBG}) \end{array} \end{aligned}$$

The fifth model $M_5$ was proposed to test the nonlinearity of duration of diabetes and it is given by

7 $$\begin{aligned} \begin{array}{llll} g(\mu _i) &{}= &{} \beta _0 + \beta _1 \, {gender} + \beta _2 \, {hypertension}\\ &{}+&{} \beta _3 \, {IT} + \beta _4\,FCV + \beta _5\,{DD}+ f_{gender}{age} \\ &{}+&{} f({HbA1c},{FBG}) +f_{{gender}}({CL}) \end{array} \end{aligned}$$

#### Estimation of parameters

Estimation of both penalized and unpenalized coefficients in the above models was done using penalized iterative reweighted least squares (PIRLS). We have used evenly spaced knots with $k=10$ in the ranges of the covariate $x_j$ for main effect and for covariate by factor interaction of the smoothing functions, and $k=8$ for the tensor product interaction [36]. Since under finite sample size, prediction error criteria, such as generalized cross validation (GCV) (for the known scale parameter) and unbiased risk estimator (UBRE) (for the unknown scale parameter), is more likely to develop multiple minima which undersmooth the function $f_j$ relative to restricted maximum likelihood (REML), therefore the smoothing parameter selection in the analyses was done using REML and data analysis was done using |gam| function from |mgcv| package in R statistical software. For the detailed information on parametric estimation and modeling of semiparametric model see [36] and for covariate by factor interaction of a smoothing function see [37].

#### Test of nonlinearity and model evaluation

The hypothesis test for a statistically significance of a nonlinear effect of a continuous covariate $x_j$ was done using the likelihood ratio test by fitting two models, that is, we fit first a model where $x_j$ has a linear relationship and then a second model with a nonlinear relationship. Then the hypothesis to be tested is, there is a linear relationship between the covariate $x_j$ and the response against there is no linear relationship between the covariate $x_j$ and the response. Following [39], model diagnostic or model evaluation was done using plots of smoothes and their standard errors. In addition, the normality assumption was tested using quantile quantile plot (Q-Q plot) and histogram.

### Results

#### Missing data imputation

**Fig. 3:**
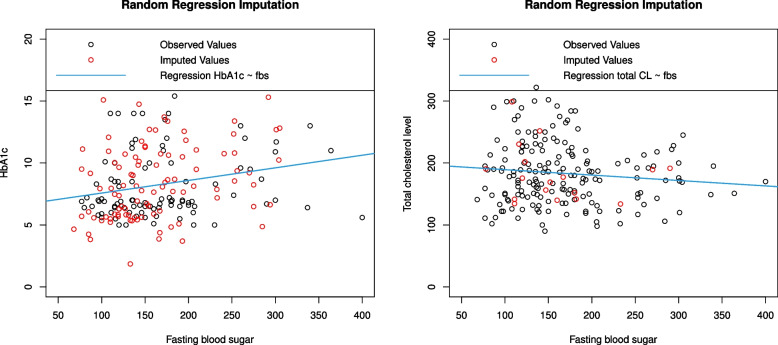
Goodness of fit of the imputed observation relative to the observed data for HbA1c and total cholesterol level

The presence of missing observations in some of the variables in a data has an effect on statistical inference, such as poor precision on confidence intervals and biased on parameter estimates, which may result poor statistical power [40]. Therefore, we imputed the missing values of variables with more than 5% missing values using multivariate stochastic regression imputation technique [41]. Furthermore, the missing observations in two variables, cholesterol level and HbA1c which had 9% and 50% missing values, respectively were imputed using the above technique. According to [42], under missing at random and missing completely at random, multivariate imputations produce unbiased estimates at a high amount of missing. Furthermore, the author also shows the bias of multivariate imputation is consistent regardless of increasing imputation from 10% to 50%. As it can be seen in Fig. 3, the distribution for the imputed values and observed values are similar.

#### Test of multicollinearity and nonlinearity

The covariates were checked for multicollinearity using the variance inflation factor (VIF) before adding them to the model. None of these VIFs (the values are between 1.08 and 1.21) were greater than 5 suggesting the collinearity is not strong to affect the statistical inference in the analysis. Next, the five proposed models in the methodology section were fitted and a likelihood ratio test was used to test the nonlinearity of continuous covariates. As it can be seen from Table 1, the deviance for testing the nonlinearity of the interaction of age by gender is 11.98 with $p-value=0.0461$, indicating that there was a significant nonlinear relationship between the odds of DR and the interaction of age by gender. Similarly, the deviance for the nonlinearity test of cholesterol level by gender is 37.20 with $p-value=0.0012$. Thus, there was a significant nonlinear relationship between the odds of DR and the interaction of cholesterol level by gender. The likelihood ratio test for the relationship between the odds of diabetic retinopathy and duration of diabetes has deviance equals to 13.02 with $p-value=0.0228$ (Table 1), therefore, the relationship was significantly nonlinear. The nonlinearity test for the interaction of HbA1c and FBG was also significant $(p-value=0.0157)$ supporting the nonlinear relationship.

**Table 1:** Test of nonlinearity for the continuous covariates

Nonlinearity test	Models	Resid.Df	Resid.Dev	Df	Deviance	$pr(>chi)$
$HbA1c\times FBG$	$M_2$	158.20	113.21			
$M_2\text{ vs } M_1$	$M_1$	153.43	99.62	4.77	13.59	$0.0157^*$
Age by gender	$M_3$	158.99	111.61			
$M_3\text{ vs } M_1$	$M_1$	153.43	99.62	5.53	11.98	$0.0461^*$
CL by gender	$M_4$	168.56	136.83			
$M_4\text{ vs } M_1$	$M_1$	153.43	99.62	15.13	37.20	$0.0012^*$
DD	$M_5$	158.41	112.64			
$M_5\text{ vs } M_1$	$M_1$	153.43	99.62	4.97	13.02	$0.0228^*$

#### Model selection and evaluation

**Fig. 4:**
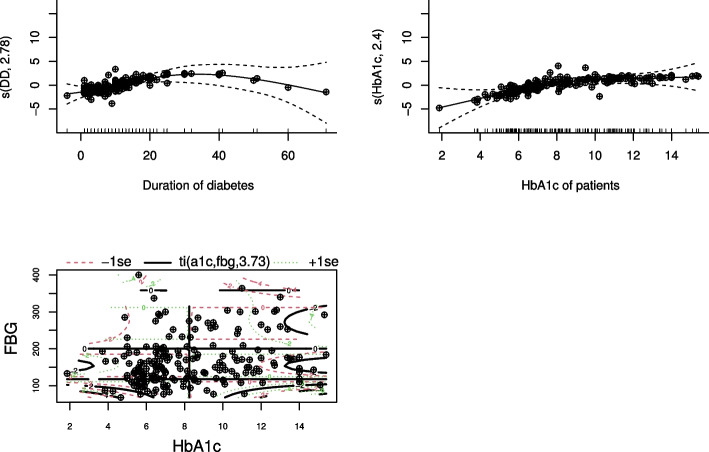
Plots of estimate of smoothes and their standard errors to check if the smoothing estimate is good

**Fig. 5:**
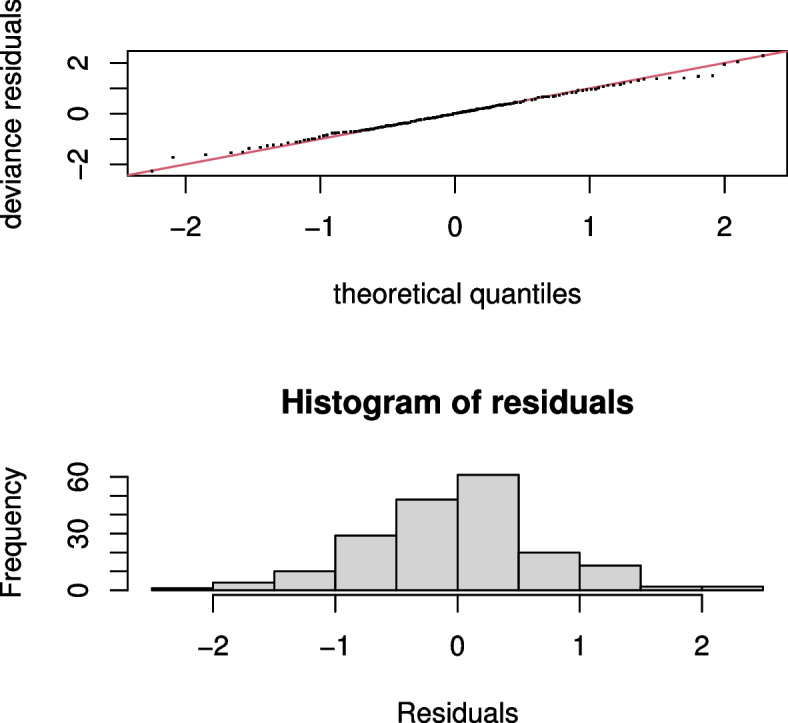
Residual plots to check the assumption of normality

In this section, we are focusing in selecting the best model which fits the data very well using Akaike’s Information Criterion (AIC). As it can be seen from Table 2, $M_1$ is a model with the smallest AIC value (163.64) which supports the nonlinearity test in Table 1. Therefore, the final model which best explains the DR data for a patient at Black Lion Hospital during the study period was $M_1$. Furthermore, The model chosen ($M_1$) was evaluated using different residual plots. For instance, the residuals in the plots of smoothes and their standard errors in Fig. 4 follow the fitted functions, indicating that the estimate of the smooth is not underestimated or overestimated. Furthermore, the Q-Q plot and the histogram in Fig. 5 show that the residuals are normally distributed. Therefore, the result in the next section is based on $M_1$.

**Table 2:** Model comparison using AIC

Models	$M_1$	$M_2$	$M_3$	$M_4$	$M_5$
AIC	163.64	169.44	166.52	176.32	168.61

#### Semiparametric multivariable analysis

**Fig. 6:**
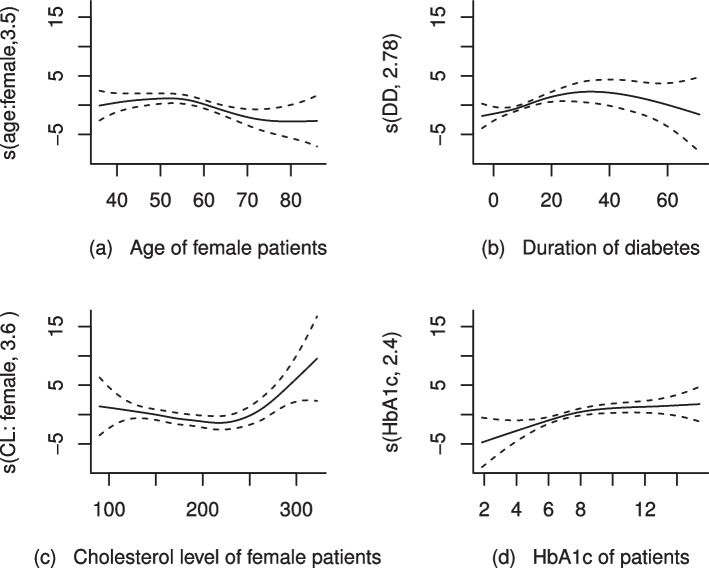
Estimate of smooths in a semiparametric model

**Fig. 7:**
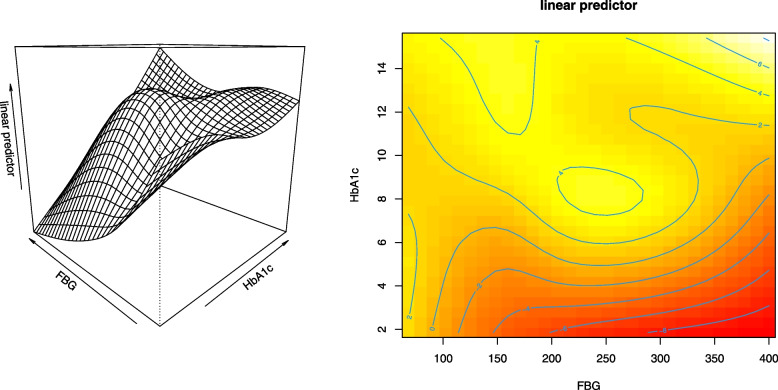
Estimated effects for the tensor product smooth interaction $HbA1c\times FBG$ in a semiparametric model

The results from fitting $M_1$ are displayed in Table 3, Figs. 6 and 7. There were a total of 191 people with type II diabetes mellitus in the study, of which 98 (51.3%) had DR (Table S1). Keeping the effects of being hypertensive, insulin treatment, frequency of clinical visit, interaction of age by gender, duration of diabetes, $HbA1c \times FBG$ and interaction of total cholesterol level by gender constant, the odds of having DR for a male patient was 3.5 (95% CI:1.14-11.09) times higher than that of female patients. Keeping the effect of other covariates constant, the odds of having DR was significantly higher for the hypertensive patient (adjusted odds ratio (AOR)=38.9, 95% CI: 9.85-153.23). The odds of having DR for a patient who used insulin treatment was 6.2 (95% CI: 1.81-13.84) times higher than the odds of having DR for a patient who did not use insulin treatment to control their blood glucose level keeping the effect of other covariates constant. Keeping the effect of other covariates constant, the odds of having DR for a patient whose clinical visit was every 3 months was 8.7 (95% CI: 2.13-35.9) times higher than the odds of having retinopathy for a patient whose clinical visit was every 1 month. Similarly, the odds of having DR was higher for a patient who had follow-up every 6 months (AOR=6.7, 95% CI: 1.63-27.41) as compared to a patient who had follow-up every one month keeping the effect of other covariates constant.

**Table 3:** Semi parametric estimate of socio-demographic and clinical variables that have a significant effect on DR

Predictors	Levels	Df	$\hat{\beta }$ (se)	$p-value$	AOR	95%CI
Intercept		1	-4.30 (0.83)	$<0.0001$		
Gender	Male	1	1.27 (0.58)	$0.0280^*$	3.5	[1.14, 11.09]
Hypertension	yes	1	3.66 (0.70)	$<0.0001$	38.9	[9.85, 153.23]
IT	yes	1	1.84 (0.63)	$0.0040^*$	6.2	[1.81, 13.84]
FCV	every 3 month	1	2.17 (0.73)	$0.0020^*$	8.7	[2.13, 35.9]
	every 6 month	1	1.91 (0.72)	$0.0080^*$	6.7	[1.63, 27.41]
Nonlinear Terms						
$f_{gender}(age)$	Female	3.2		$0.0357^*$		
$f_{gender}(age)$	Male	1.0		0.2386		
f(DD)		2.7		$0.0059^*$		
$f_{gender}(CL)$	Female	3.6		$0.0166^*$		
$f_{gender}(CL)$	Male	3.7		0.1321		
*f*(*HbA*1*c*)		2.3		$0.0020^*$		
*f*(*FBG*)		1.0		0.2784		
f(HbA1c, FBG)		3.7		$0.0500^*$		

The result in Table 3 illustrates that holding the effects of other covariates constant, there was a significant nonlinear relationship between the log odds of having DR and age of female patients ($p-value=0.0357$) with estimated degrees of freedom 3.2. Furthermore, visual inspection of Fig. 6(a) shows that the log odds of having DR for female patients increase slightly with age at the begning, but it shows a gradual decline after the age of 65 years: the confidence band is very wide in this age range, it could be because of a few number of patients older than 65 years who had DR. Table 3 also reveals that, duration of diabetes had a significant nonlinear relationship $(p-value=0.0059)$ with the log odds of having DR. Moreover, according to Fig. 6(b), the functional relationship between duration of diabetes and log odds of having DR looks inverted U-shape with estimated degrees of freedom 2.7. However, the confidence band after 30 years of duration of diabetes becomes notably wider, indicating greater variability which may be due to a small number of observations in that interval. As it can be seen in Fig. 6(c), the finding of this study also indicates that the relationship between the log odds of having DR and female cholesterol level was initially flat, but a moderate increment in the log odds of having DR for a female patient was observed for a total cholesterol level $>250mg/dL$. Table 3 also shows that there was a significant nonlinear relationship between the log odds of having DR and female cholesterol level ($p-value= 0.0166$) with degrees of freedom 3.6. Similarly, there was a significant nonlinear relationship between the log odds of having DR and HbA1c $(p-value=0.0020)$ with estimated degrees of freedom 2.3. As it can be seen in Fig. 6(d), the log odds of having DR has an increasing pattern when the patient HbA1c is between $6\%-11\%$ and flat pattern was observed for HbA1c greater than $11\%$. However, the confidence band at the initial (for HbA1c between (0-4)%) and at the end (for HbA1c > 11%) was wide, which may be due to greater variability at these intervals.

There was a significant nonlinear relationship between the log odds of having DR and $HbA1c \times FBG$ $(p-value=0.0500)$ with degrees of freedom 3.7 (Table 3). The 3D contour plot in Fig. 7 indicates that, the risk of DR increases with increasing HbA1c slowly for the patient with low FBG and the risk was higher for high FBG-HbA1c combinations. Furthermore, the 2D contour plot also shows that the risk of DR was higher when both FBG and HbA1c were simultaneously high. The darker red region indicates that the risk of being DR was minimum for the low percentage of HbA1c. Moreover, the combination of $HbA1c \ge 6\%$ and $FBG \ge 150mg/dL$ shows a relatively high risk of DR. Generally, the distribution of numerical values (value of linear predictor) on the contour lines in the three regions; dark red (low risk), light red (intermediate risk), and yellow (high risk) of the plots tell the nonlinear relationship between the linear predictor measuring the risk of DR and $HbA1c \times FBG$.

### Discussion

This study was aimed to identify the risk factors of DR using data collected from Black Lion Hospital at Addis Ababa, Ethiopia. In the current study, rather than using statistical methods which impose some parametric assumptions, we focused on the data-driven relationship. The results from applying semiparametric regression analysis on the data showed that the odds of having DR had a significant linear association with gender, hypertension, insulin treatment and frequency of clinical visit. In addition, the log odds of having DR had a significant nonlinear association with the interaction of age by gender (for female patients), duration of diabetes, interaction of cholesterol level by gender (for female patients) and the interaction of HbA1c by FBG. Since several studies [3, 4, 13, 43, 44] discussed the linear effects of gender, hypertension, insulin treatment and frequency of clinical visit on the odds of having DR, in this section we focused on the nonlinear terms of the findings.

In a nonlinear terms of a semiparametric analysis, some interaction terms were incorporated based on scientific literature and exploratory analysis, i.e., age by gender, cholesterol level by gender, and the tensor product or interaction of HbA1c and FBG. As it was discussed in the Result section, almost half of the study participants had DR. Therefore, understanding both additive and interaction effects of those socio-demographic and clinical variables is crucial to prevent the progression of DR. The result of this study show that the log odds of having DR have a significant nonlinear relationship with the age of female patients. However, sex and age based stratified analysis showed that the incidence rate of sight-threatening DR had a decreasing trend for women as compared to men [45]. Despite this, several studies reported the marginal effect of age and gender on being DR [15, 20, 46]. However, these literatures show linear relationship between the odds of having DR with age and gender.

In the current study, the duration of diabetes since a patient confirmed type II diabetes was appeared as one of the risk factors of DR. We identified a nonlinear relationship between duration of diabetes and the log odds of having DR. This result agrees with previous studies [3, 12, 14, 15, 17]. However, these studies used a generalized linear model which can only identify a linear association between duration of diabetes and linear predictor rather than using a data-driven relationship like a semiparametric model. Furthermore, the interaction between total cholesterol level and gender had a significant nonlinear association with the log odds of having DR. Though, Hanai et al. [47] investigated the progression of diabetic kidney disease and found that those lipid profile parameters are correlated with gender as a predictor of kidney disease progression. Further, Kaewput et al. [48], conducted a nationwide cross-sectional study in Thailand showing that DR had a significant association with renal function. Therefore, these two studies indirectly revealed that the interaction between the lipid profile of a patient and gender had a significant effect on being DR. However, the results of these studies did not show the level of cholesterol that a patient (he/she) could be at high risk of DR.

The other interesting finding of our study was the significant nonlinear relationship between interaction $HbA1c \times FBG$ and the log odds of having DR. Despite the nonlinear relationship, a semiparametric model based on the tensor product of HbA1c and FBG suggested that the combination of a high level of HbA1c and a high level of FBG resulted in a higher risk of being DR. Our study finding agrees with a study that used 10-year follow-up data [22]. Their finding suggested that patients with DR at the baseline had a high level of FBG and a high level of HbA1c. However, our finding contradicts some of the previous studies. For example, Gimeno-Orna et al. [21] conducted a cohort study with a mean follow-up period of 5.2 years to examine whether FBG variability determines the onset of DR irrespective of HbA1c. Their finding from univariate logistic regression analysis showed that the interaction of mean HbA1c and FBG variability was not a significant risk factor of DR. However, this result may be due to the nonlinear relationship between linear predictor for the interaction $HbA1c \times FBG$ and DR. Besides, several studies showed the marginal effects of FBG and HbA1c on DR [12, 14, 20].

### Conclusion

This study identified the possible risk factors of DR based on data obtained from BLH using a semiparametric model. The results from this study indicate that clinical variables related to patient characteristics were strong predictors of DR. The results of the semiparametric analysis reveal evidence that being hypertensive, insulin treatment, 3 and 6-months clinical visits were strong predictive factors of DR. Moreover, duration of diabetes, interaction of age by gender, and cholesterol level by gender had significant nonlinear relationships with DR. Additionally, the nonlinear relationship between the interaction $HbA1c \times FBG$ and the linear predictor suggested that the risk of DR was higher when the value of both HbA1c and FBG high. The nonlinear relationship between DR and continuous clinical predictor can help health professionals to understand about the nature of the predictor and it’s relation with the outcome. This will help them to identify if a given patient is at high risk of DR or not.

Based on the findings we recommend that health care professionals should give more attention to the possible effect of clinical variables which can lead people with a type II diabetes mellitus to DR. Furthermore, the researchers should assess the type of relationship between DR and continuous clinical variables using exploratory analysis before introducing them to a statistical model as this may affect results of their analysis and hence a conclusion of their findings. Finally, since our study was based on one hospital, we recommend that a similar study should be conducted across the country to get more information to improve intervention strategies.

## Supplementary Information

**Additional file 1: Table S1.**

## Data Availability

The data sets used and/or analysed during the current study are available from the corresponding author on reasonable request.

## Abbreviations

AIC: Akaike information criteria
BLH: Black lion hospital
CL: Cholesterol level
DD: Duration of diabetes
DM: Diabetes Mellitus
DR: Diabetic retinopathy
FBG: Fasting blood Glucose
FPG: Fasting plasma glucose
FCV: Frequency of clinical visits
GCV: Generalized cross validation
HbA1c: Hemoglobin A1c
IDF: International diabetes federation
IT: Insulin treatment
NPDR: Non-proliferative diabetic retinopathy
PDR: Proliferative diabetic retinopathy
PIRLS: Penalized iterative reweighted least squares
REML: Restricted maximum likelihood
VIF: Variance inflation factor
UBRE: Unbiased risk estimator
