# Supplementary material for: Semiparametric modelling of diabetic retinopathy among people with type II diabetes mellitus
Source: BMC Med Res Methodol. 2023 Jan 9;23:7. doi: 10.1186/s12874-022-01794-4 (PMC9830762; doi:10.1186/s12874-022-01794-4)
Supplement: Supplementary file 1 — Additional file 1: Table S1. [file 12874_2022_1794_MOESM1_ESM.pdf]

S0.0.1 Descriptive statistics

There were a total of 191 type II diabetic patients in the study, of which 98 (51.3%) had DR. Among the total sample, 114 (59.7%) were female, of which 51 (26.7%) of them had DR. More than half (54.8%) of the total patients in the study were hypertensive, of which 76 (39.8%) of them had DR. Out of patients who used insulin treatment, 40 (20.9%) of them had DR. The total number of patients whose clinical visit was within 1 month interval was 43 (22.6%), of which 7.4% of them had DR. Moreover, of those patients whose clinical visit were within 3 and 6 months interval, 38 (20%) and 46 (24.2%) of them had DR, respectively. The mean age of patients with DR was 58.5 years with a standard deviation of 10.1 years (Table S1). The average diabetic duration since a patient was confirmed to type II diabetes mellitus till the start of the study period was 15.2 years with a standard deviation of 10.9 years. In this study, HbA1c is measured in percentage form known as Diabetes Control and Complications Trial (DCCT) units. The mean HbA1c for a patient with DR was 9.3% with a standard deviation of 2.8%. In addition, the average total cholesterol level for a patient who had DR was 187.6 mg/dL. The mean FBG for a patient with DR was 170.9 mg/dL with a standard deviation of 66 mg/dL (Table S1).

As it can be seen from the box plot in Figure 2, the median total cholesterol level for female was around 186 mmol/L and the median cholesterol level for male patient was around 171 mmol/L.

Table S1: Summary statistics of diabetic retinopathy status of type II diabetic patients vs socio-demographic and clinical variables

		Diabetic Retinophaty(DR)		
Characteristics	Levels	Patient with DR,N(%)	Patient without DR, N(%)	Total
Gender	M	47 (24.6)	30 (15.9)	77 (40.5)
	F	51 (26.7)	63 (32.8)	114 (59.5)
Hypertension	No	22 (11.5)	63 (32.9)	85 (44.4)
	Yes	76 (39.8)	30 (15.8)	106 (54,8)
IT	No	58 (30.4)	79 (41.4)	137 (71.7)
	Yes	40 (20.9)	14 (7.3)	54 (28.3)
FCV	every 1 month	14 (7.4)	29 (15.2)	43 (22.6)
	every 3 month	38 (20.0)	34 (17.9)	72 (37.9)
	every 6 month	46 (24.2)	29 (15.3)	75 (39.5)
		mean(sd)	mean(sd)	$n = 191$
Age		58.5(10.1)	56 (10.4)	
DD		15.2(10.9)	9.2 (8.8)	
HbA1c		9.3 (2.8)	7.09 (2.3)	
CL		187.6(56.2)	179.3(41.5)	
FBG		170.9 (66.0)	160.7 (60.6)	
